# Stress resilience in plants: the complex interplay between heat stress memory and resetting

**DOI:** 10.1111/nph.20377

**Published:** 2025-01-23

**Authors:** Tobias Staacke, Bernd Mueller‐Roeber, Salma Balazadeh

**Affiliations:** ^1^ Institute of Biology Leiden, Sylvius Laboratory Leiden University Sylviusweg 72 Leiden 2333 BE the Netherlands; ^2^ Institute of Biochemistry and Biology University of Potsdam Karl‐Liebknecht‐Straße 24‐25, Haus 20 Potsdam 14476 Germany

**Keywords:** heat stress acclimation, memory, molecular mechanisms, plants, resetting

## Abstract

Heat stress (HS) poses a major challenge to plants and agriculture, especially during climate change‐induced heatwaves. Plants have evolved mechanisms to combat HS and remember past stress. This memory involves lasting changes in specific stress responses, enabling plants to better anticipate and react to future heat events. HS memory is a multi‐layered cellular phenomenon that, in addition to epigenetic modifications, involves changes in protein quality control, metabolic pathways and broader physiological adjustments. An essential aspect of modulating stress memory is timely resetting, which restores defense responses to baseline levels and optimizes resource allocation for growth. Balancing stress memory with resetting enables plants to withstand stress while maintaining growth and reproductive capacity. In this review, we discuss mechanisms and regulatory layers of HS memory and resetting, highlighting their critical balance for enhancing stress resilience and plant fitness. We primarily focus on the model plant *Arabidopsis thaliana* due to the limited research on other species and outline key areas for future study.

**Table:** 

	Content	
	Summary	2402
I.	Introduction	2402
II.	Mechanisms of heat sensing in plants	2403
III.	Plant strategies for combatting HS: from heat stress response to memory and resetting	2404
IV.	Mechanisms of HSM	2405
V.	Mechanisms of HS resetting	2411
VI.	Tissue‐specific responses to HSM: role of the shoot apex	2413
VII.	Future perspectives	2415
	Acknowledgements	2416
	References	2417

## I. Introduction

Increases in greenhouse gases raise atmospheric energy levels, generating higher mean annual temperatures in many parts of the world. Regional warming is expected to exceed the global average of +2.8°C by 2100, likely prompting more frequent and severe heat waves (IPCC, 2021). Agriculture is particularly vulnerable to the impact of climate change (Malhi *et al*., 2021). Predictions indicate that every 1°C increase in global mean temperature could reduce yields of major crops, for example maize (−7.4%), wheat (−6.0%), rice (−3.2%) and soybean (−3.1%) (Zhao *et al*., 2017). Therefore, enhancing the resilience of crops against heat stress (HS) is essential for ensuring food security.

Plants elicit specific adaptive responses to changes in temperature with varying exposure durations. When exposed to moderately elevated temperatures, plants accelerate processes, such as hypocotyl elongation, petiole elongation and leaf hyponasty, moving aboveground organs away from the soil's heat. These responses also hasten the transition from vegetative to reproductive development and are collectively known as thermomorphogenesis (Gray *et al*., 1998; Quint *et al*., 2016; Vu *et al*., 2019).

During excessively high temperatures (HS), plants experience cellular damage, primarily due to impaired photosynthesis and respiration, the accumulation of misfolded proteins and the formation of reactive oxygen species (ROS) (Larkindale & Knight, 2002; Kotak *et al*., 2007; Mittler *et al*., 2012; Hasanuzzaman *et al*., 2013). The developmental stage when plants experience heat also affects their survival. Young seedlings are particularly vulnerable due to their small size, proximity to hot soil and lack of fully developed organs, such as roots, to mitigate the effects of excessive water loss (Jagadish *et al*., 2021). Reproductive tissues present during flowering and gametogenesis are also susceptible to HS, leading to reduced fertility, poor seed set and, ultimately, significant yield losses (De Storme & Geelen, 2020; De Jaeger‐Braet *et al*., 2022).

Substantial progress has been made in identifying the physiological and molecular responses of crops and model plant species to moderately high temperature or acute HS (Quint *et al*., 2016; Ohama *et al*., 2017; Casal & Balasubramanian, 2019; Haider *et al*., 2021; X. Wang *et al*., 2023). However, in natural environments, plants often face multiple recurring HS events rather than a single occurrence. The timing of HS episodes can vary widely: subsequent events may occur shortly after the previous one or much later. Our understanding of how plants cope with such repetitive HS remains limited. Constitutive upregulation of stress tolerance mechanisms often results in reduced plant growth, which can be detrimental to overall plant fitness (Liu *et al*., 1998; Sakuma *et al*., 2006; Ogawa *et al*., 2007; Zhu *et al*., 2009; Ikeda *et al*., 2011). However, while plants reset certain stress‐induced changes after the stress has subsided, they retain specific beneficial modifications that enhance their ability to respond more quickly and effectively to future stresses – a phenomenon known as acquired tolerance. When this adaptive capacity persists for an extended period, it is referred to as ‘stress memory’. Studies suggest that both stress memory and resetting mechanisms are actively regulated processes. Understanding these mechanisms is key to developing plants with a balanced capacity for memory and resetting, ensuring their survival and growth in the challenging conditions of climate change. Here, we review current knowledge about the regulation of HS memory (HSM or thermomemory) in plants and how it is reset, and highlight aspects requiring further investigation.

## II. Mechanisms of heat sensing in plants

Plants cannot move away from unfavorable environmental conditions and require sophisticated mechanisms for sensing and responding to stress. An ongoing question in HS research is how plants perceive high‐temperature signals and convert them to cellular responses. Already identified mechanisms for sensing warming temperatures and HS were extensively reviewed (Mittler *et al*., 2012; Jung *et al*., 2016, 2020; Vu *et al*., 2019; Lamers *et al*., 2020; Delker *et al*., 2022; Kerbler & Wigge, 2023; Casal *et al*., 2024). These include changes in plasma membrane composition and fluidity, altered folding, mobility and activity of membrane‐associated proteins, for example calcium channels, and release of cytoplasmic calcium (Ca^2+^) ions linked to subsequent activation of downstream signaling (Mittler *et al*., 2012). Moreover, increased levels of reactive oxygen and nitrogen species appear to participate in thermal perception (Volkov *et al*., 2006; Mittler *et al*., 2012; He *et al*., 2022).

In response to changes in ambient temperature, light receptors like PhyB (Jung *et al*., 2016; Legris *et al*., 2016), transcription factors, such as PHYTOCHROME‐INTERACTING FACTOR 4 (PIF4) and PIF7 (Koini *et al*., 2009; Kumar *et al*., 2012; Chung *et al*., 2020), and histone H2A.Z‐containing nucleosomes (Kumar & Wigge, 2010) are involved in temperature‐induced changes in growth and development (Delker *et al*., 2022). Additionally, the circadian clock component EARLY FLOWERING 3 (ELF3) acts as a sensor of elevated temperatures in Arabidopsis. Under normal ambient temperature, ELF3 suppresses temperature‐responsive genes (including *PIF4*) in a complex with ELF4 and LUX ARRHYTHMO (LUX). However, this suppression is reversed at increased ambient temperature, illustrating ELF3's dynamic role (Box *et al*., 2015; Silva *et al*., 2020). Two models describe a mode‐of‐action for ELF3: at low ambient temperature (17°C), ELF3 in root cells is dispersed in the nucleus, whereas at higher temperatures (27°C and 35°C), it forms subnuclear speckles through phase separation driven by its prion domain, leading to alleviation of repression on target genes (Jung *et al*., 2020). Another model proposes that under ambient temperature (22°C), ELF3 localizes in subnuclear foci in root and hypocotyl cells to repress target genes, but this localization is disrupted under warmer conditions (27°C) (Ronald *et al*., 2021). These differences suggest that ELF3's complex role as a thermosensor is influenced by cellular contexts and experimental conditions, requiring further exploration (Murcia *et al*., 2022).

A recent study by Bohn *et al*. (2024) revealed that THERMO‐WITH ABA‐RESPONSE 1 (TWA1) is a novel protein essential for an early response to heat. TWA1 was identified in a screen for thermotolerance in abscisic acid (ABA) hypersensitive mutants. A *twa1‐1* knockout mutant exhibited a markedly higher induction of an ABA‐responsive luciferase reporter than the wild‐type (WT) at elevated temperatures. TWA1 is an intrinsically disordered protein with a highly variable N‐terminal region (20 amino acids) crucial for its thermosensory function. At elevated temperatures (30°C and 35°C), TWA1 undergoes a temperature‐dependent conformational change. It accumulates at specific nuclear subdomains, where it forms a repressor complex with the JASMONATE‐ASSOCIATED MYC‐LIKE (JAM2) transcription factor and TOPLESS (TPL) and TOPLESS‐RELATED (TPR) proteins. This complex is essential for the rapid activation of heat‐responsive genes like *HEAT SHOCK FACTOR A2* (*HSFA2*), *HEAT SHOCK PROTEIN 21* (*HSP21*), *HSP26.5* and *HSP17.6* (Bohn *et al*., 2024). HSFA2 is a key regulator of HSM (to be described later), but it is still uncertain whether TWA1's sensory function is essential for HSFA2‐mediated HSM.

Another recently reported thermosensory mechanism involves nitric oxide (NO) as a signaling molecule, which originates at the shoot apex, travels to other plant organs and activates *HSFA2* expression via the GT‐1 transcription factor to trigger a response to HS (He *et al*., 2022) (see Section VI). This study highlights the importance of tissue‐specific heat responses and the need for further research into how different heat sensors integrate signals across tissues.

## III. Plant strategies for combatting HS: from heat stress response to memory and resetting

### 1. From HSR to HSM

**Fig. 1:**
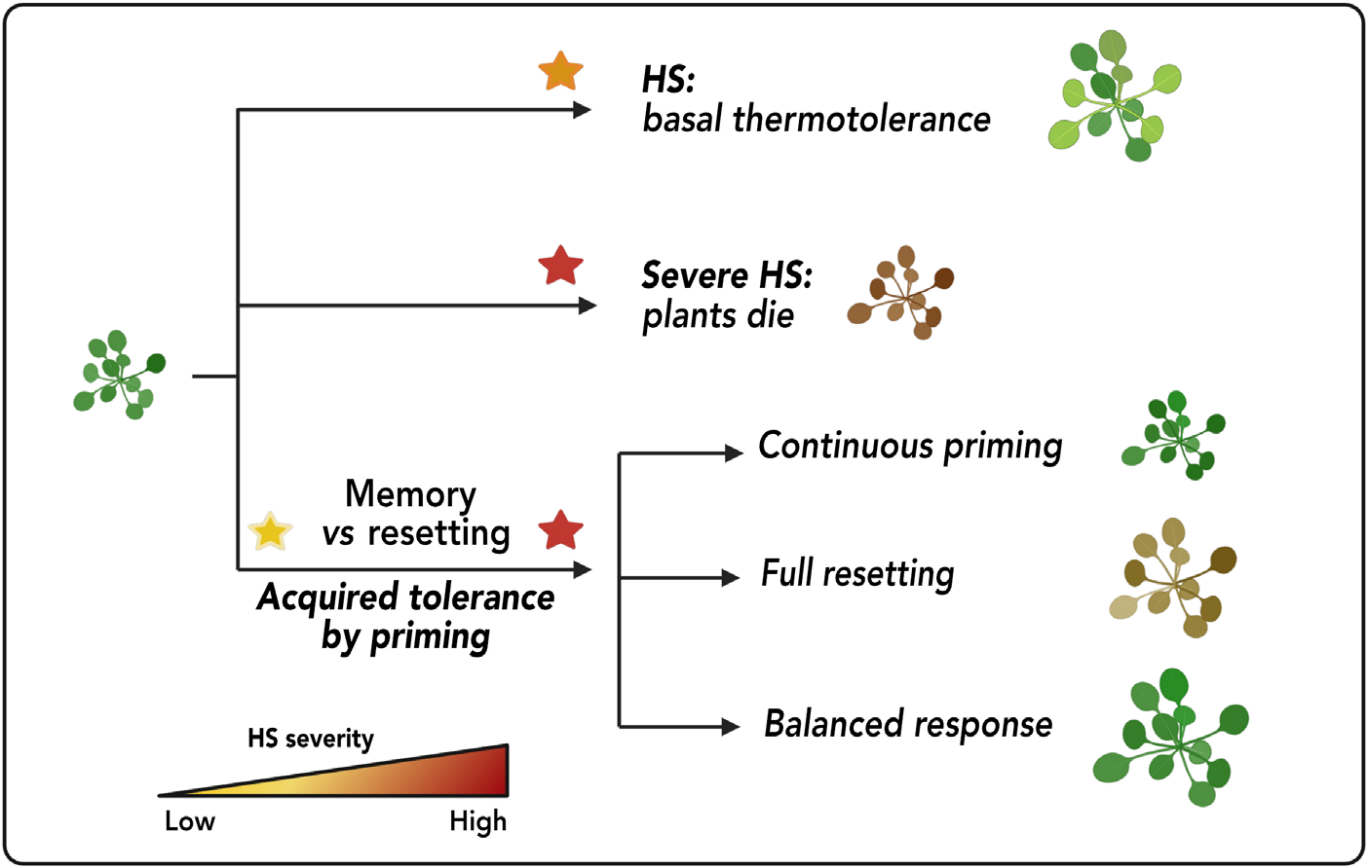
Model depicting plant responses to heat stress (HS) and the development of acquired thermotolerance through priming. Plants have an inherent ability, called basal thermotolerance, which allows them to withstand certain levels of heat stress (HS; represented by the orange star). However, when the severity of HS surpasses the plant's threshold, indicated by the red star, it results in plant death, while plants that have undergone priming (marked by the yellow star) exhibit different and more robust responses to subsequent severe HS. After priming, during the interval between the initial priming and a subsequent HS event, plants establish a form of stress memory. This memory involves selectively retaining certain stress‐related changes that are advantageous for future stress responses while resetting others for normal growth. Achieving a balance between maintaining stress memory and resetting these changes is vital for the plant's survival, growth and productivity. Full resetting may impair the plant's ability to respond to subsequent stress, whereas continuous activation of all priming responses could hinder growth. The figure depicts three possible responses to HS after priming: continuous priming, leading to sustained readiness against future stress; full resetting, which returns the plant to its original prestress state; and a balanced response, allowing optimal recovery while retaining some level of readiness. The triangular gradient in the figure represents increasing HS severity from low to high. Created in BioRender. Bz, S. (2025) https://BioRender.com/n88r065.

When exposed to excessively elevated temperatures, plants, like other organisms, activate a heat stress response (HSR). This involves induction of genes that produce molecular chaperones, including heat shock proteins (HSPs) and antioxidants, and other mechanisms vital for maintaining protein homeostasis and cellular integrity (Wang *et al*., 2004; Scharf *et al*., 2012). The HSR is an evolutionary conserved process primarily orchestrated by DNA‐binding heat shock transcription factors (HSFs) (Bakery *et al*., 2024). HSFs are numerous in plants (typically > 20), but fewer (about one to four) exist in other organisms like baker's yeast, fruit fly and vertebrates, demonstrating a more sophisticated regulatory system in plants (von Koskull‐Döring *et al*., 2007; Akerfelt *et al*., 2010; Scharf *et al*., 2012). Among HSFs, class A1s are key regulators of the HSR in plants (Mishra *et al*., 2002; Liu *et al*., 2011; Yoshida *et al*., 2011). HSFA1s are continuously expressed, but their activity is intricately regulated at the protein level to ensure a rapid response to HS. A key regulatory mechanism is the heat‐induced release and activation of HSFA1s from suppression by the HSP70/90 complex, which facilitates their rapid transfer to the nucleus (Ohama *et al*., 2016, 2017). This activation is essential for triggering HS‐responsive genes, including activation of other TFs, for example HSFA2, DREB2A and MBF1C (Yoshida *et al*., 2011). However, elements of the HSR are also regulated through pathways independent of HSFA1s, indicating a complex network of regulatory mechanisms beyond the HSFA1‐dependent pathway (Wu *et al*., 2012; Ohama *et al*., 2017; Huang *et al*., 2021). While HSR is a core mechanism, plants adapt to varying HS conditions through distinct responses, each activating the HSR differently. This variability depends on factors like the duration and intensity of heat exposure, as well as previous stress experiences (Fig. 1). Basal thermotolerance, an inherent ability of plants, enables them to combat sudden HS by rapidly activating a HSR. If heat surpasses the basal tolerance limit, it causes significant damage at molecular, physiological and cellular levels, potentially leading to plant death. However, if plants experience a milder and transient HS, it can act as a priming stimulus, enabling them to acquire thermotolerance (or heat acclimation). Consequently, plants become better prepared to withstand a subsequent severe and potentially lethal HS (often referred to as triggering HS). The concept of priming has been recognized for decades, and numerous environmental and chemical factors have been demonstrated to prime plants for improved stress resistance (Conrath, 2009; Ellouzi *et al*., 2013; Savvides *et al*., 2016; Kerchev *et al*., 2020). Priming triggers widespread changes across molecular, metabolic, physiological and cell‐biological levels, resulting in a new state of homeostasis (Crisp *et al*., 2016; Hilker *et al*., 2016; Serrano *et al*., 2019). This altered homeostasis enhances the HSR, allowing plants to react more quickly or robustly to subsequent stress.

Priming and subsequent triggering (severe) stress do not necessarily occur in close succession; rather, priming can leave a lasting imprint that persists even after the priming stimulus has vanished, thereby creating a molecular ‘stress memory’ that enables plants to maintain enhanced resilience over an extended period (Ding *et al*., 2013; Lämke *et al*., 2016; Charng *et al*., 2023). For instance, in Arabidopsis, some HSR genes show transcriptional memory. This includes sustained altered expression after the priming stimulus has faded (type I), or a faster or enhanced transcriptional response to subsequent HS following the initial priming stimulus (type II) (Bäurle & Trindade, 2020). HSFA2, triggered by HSFA1s, plays a crucial role in the establishment and maintenance of this memory through its interaction with other HSFs and its influence on histone modifications (Lämke *et al*., 2016; Friedrich *et al*., 2021). In addition to transcriptional memory, patterns of stress memory may occur at the protein and metabolic levels (Serrano *et al*., 2019; Schulze *et al*., 2021; Mikulski & Santos‐Aberturas, 2022).

The duration of stress memory in organisms can vary widely. When stress memory lasts only within a single generation, it is often referred to as somatic memory (Bäurle & Trindade, 2020). In Arabidopsis, HSM within plant tissues and seedlings typically lasts for up to 7 d, which enables plants to respond more efficiently to subsequent heat exposures and resume growth and development after the stress period ends (Stief *et al*., 2014; Sedaghatmehr *et al*., 2016; Olas *et al*., 2021; Sharma *et al*., 2022). The underlying mechanisms are evident at multiple levels, extending beyond merely epigenetic factors (Balazadeh, 2022). While a relatively short‐term HSM may not directly address long‐term climate shifts, it may enhance an organisms' survival in the increasingly frequent and rapidly changing weather conditions associated with climate change. Somatic memory can also persist longer and span different developmental stages (Wang *et al*., 2012, 2014; Liu *et al*., 2017; Fan *et al*., 2018). However, this aspect of HSM has less often been researched. Fan *et al*. (2018) showed that heat priming during stem elongation and booting in wheat significantly enhances heat tolerance during grain filling, thereby reducing yield loss. Experimental evidence also indicates that a largely epigenetic HSM can be inherited by offspring – referred to as intergenerational (affecting the F1 generation) or transgenerational (affecting F2, F3 generations) memory (Whittle *et al*., 2009; Liu *et al*., 2019, 2021; Gallusci *et al*., 2023). In this review, we focus on mechanisms of somatic memory, which has been the subject of increasing research in recent years.

### 2. Stress memory vs stress resetting: impact on plant growth

Stress responses are generally associated with reduced growth, mainly due to redirection of metabolic and energetic resources toward maintaining tolerance mechanisms. The continuous production of stress‐induced proteins and metabolites, such as HSPs, osmoprotectants and antioxidants, is energy‐intensive, impeding growth. Experimental evidence supports this: ectopic (over‐)expression of stress regulators, including those central to the HSR (Liu *et al*., 1998; Sakuma *et al*., 2006; Ogawa *et al*., 2007; Yoshida *et al*., 2008; Zhu *et al*., 2009; Ikeda *et al*., 2011), often results in growth reduction even under nonstress conditions. Although stress memory differs from constitutive stress responses by being an induced state arising after initial stress exposure (priming), its persistence and intensity may also strongly affect plant physiology and energy allocation (Bruce *et al*., 2007; Crisp *et al*., 2016). The retention of epigenetic marks on stress‐related genes, sustained induction of gene expression or maintenance of stress‐induced proteins and metabolites during recovery phases where stress is absent can lead to trade‐offs, particularly if these processes occur during energy‐intensive growth stages. In this context, stress resetting, that is an organism's ability to recover and return to a new state of homeostasis, is a crucial counterbalance for optimizing growth under nonstress conditions (Fig. 1). While crops engineered or bred for enhanced stress memory can better withstand harsh conditions, they also need timely and effective resetting mechanisms to sustain productivity. To effectively balance stress memory and stress resetting, it is crucial to gain a deeper understanding of how these mechanisms interact across different growth stages, considering both their timing and duration.

## IV. Mechanisms of HSM

**Fig. 2:**
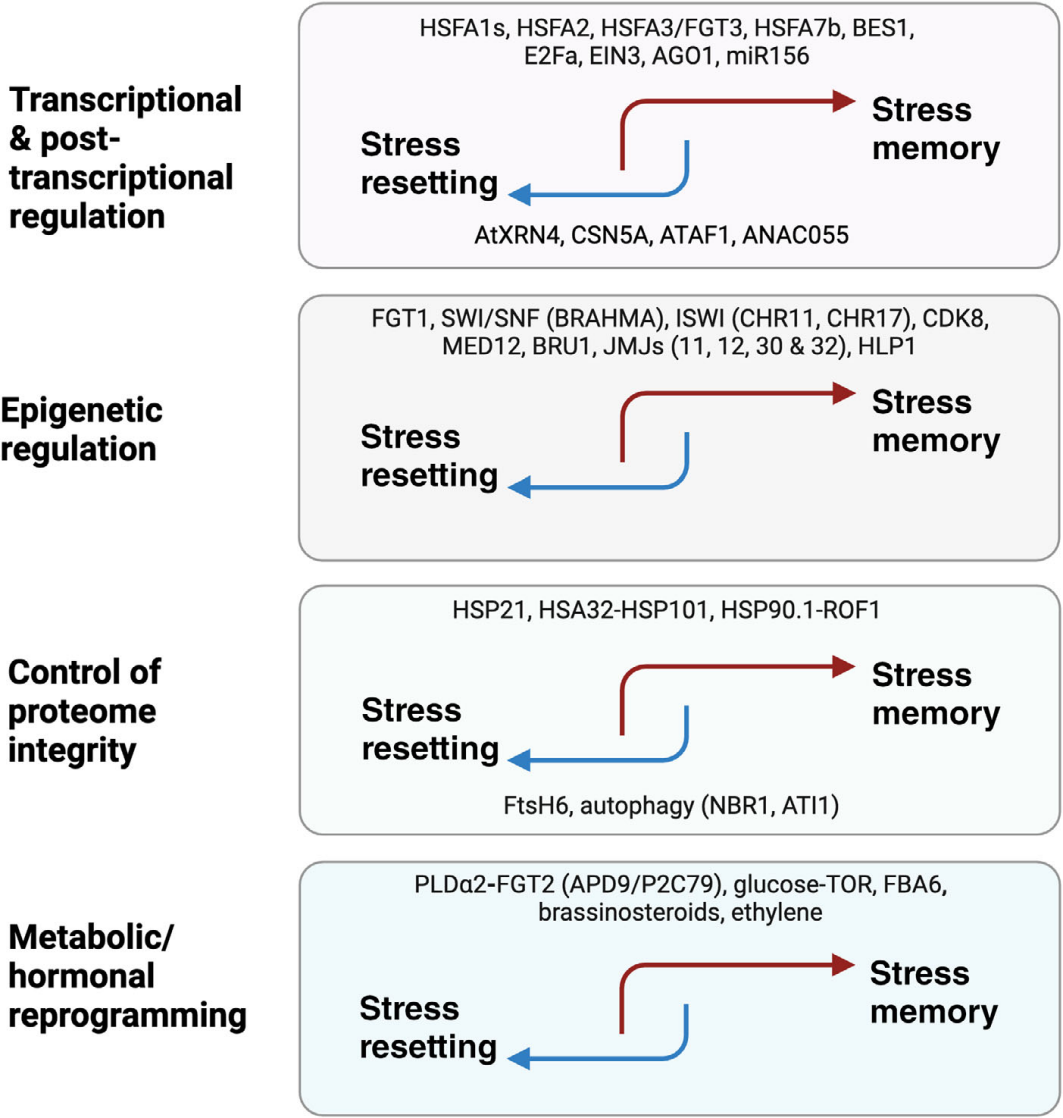
Major components involved in heat stress memory (HSM) and resetting across different regulatory levels. This diagram categorizes the molecular components associated with either stress memory (red arrows) or stress resetting (blue arrows) in four distinct layers of regulation in Arabidopsis: transcriptional and post‐transcriptional regulation, epigenetic regulation, control of proteome integrity and metabolic/hormonal reprogramming. Specific components are listed under each category to illustrate their role in stress response mechanisms. It is important to note that components within each layer may influence other regulatory levels as well. HSFA1s, Heat Shock Factor A1s; HSFA2, Heat Shock Factor A2; HSFA3, Heat Shock Factor A3; FGT3, FORGETTER 3; HSFA7b, Heat Shock Factor A7b; BES1, BRI1 EMS‐SUPPRESSOR 1; E2Fa, transcription factor E2Fa; EIN3, ETHYLENE INSENSITIVE 3; AGO1, ARGONAUTE1; miR156, microRNA156; AtXRN4, Arabidopsis 5'‐3' EXORIBONUCLEASE 4; CSN5A, CONSTITUTIVE PHOTOMORPHOGENESIS 5A; ATAF1, Arabidopsis NAC (NAM, ATAF and CUC) domain‐containing protein ATAF1; ANAC055, Arabidopsis NAC (NAM, ATAF and CUC) transcription factor 055; FGT1, FORGETTER 1; SWI/SNF (BRAHMA), SWItch/Sucrose Nonfermenting with its component BRAHMA; ISWI (CHR11, CHR17), Imitation SWItch with its components Chromatin Remodeling Protein 11 and 17; CDK8, CYCLIN‐DEPENDENT KINASE 8; Med12, MEDIATOR 12; BRU1, Brushy1; JMJs (11, 12, 30 & 32), JUMONJI demethylases 11, 12, 30 and 32; HLP1, HIKESHI‐LIKE PROTEIN 1; HSP21, Heat shock protein 21; HSA32, Heat Stress‐Associated 32 kDa Protein; HSP101, Heat shock protein 101; HSP90‐1, Heat shock protein 90‐1; ROF1, ROTAMASE 1; FtsH6, Filamentous Temperature‐Sensitive H6; NBR1, NEXT‐TO‐BRCA1; ATI1, ATG8‐INTERACTING PROTEIN 1; PLDα2, PHOSPHOLIPASE D ALPHA 2; FGT2, FORGETTER 2; APD9, Arabidopsis PP2C Clade D9 protein; P2C79, protein phosphatase 2C 79; TOR, TARGET OF RAPAMYCIN; FBA6, FRUCTOSE‐BISPHOSPHATE ALDOLASE 6. Created in BioRender. Bz, S. (2025) https://BioRender.com/c08l459.

In the last decade, multiple cellular mechanisms of HSM in plants have been discovered (Fig. 2).

### 1. Transcriptional and post‐transcriptional regulation

Early reports about the acquisition of thermotolerance in plants are from the early 1960s (Yarwood, 1961) and the 1980s (Lin *et al*., 1984), but for many years, our molecular understanding of the regulation of HSM remained limited. Pioneering work toward the elucidation of the molecular mechanisms controlling HSM was published in 2006, revealing the importance of Heat Stress‐Associated 32 kDa Protein (HSA32) in the process (Charng *et al*., 2006). This work was then extended by discovering the central role of the transcription factor HSFA2 in HSM (Charng *et al*., 2007). Knockout mutants of *HSFA2* lost the ability to endure repeated HS and showed a faster decline (than WT) in transcript levels of several HSPs, including *HSP25.3‐P* (*HSP21*), *HSP18.1* and *HSA32*, after recovery from HS. Although transcript levels were similar in mutants and WT plants during the first 2 h postpriming, they dropped rapidly in mutants after 4 h, highlighting HSFA2's role in maintaining a prolonged response. HSFA3/FORGETTER3 (FGT3), identified by a forward genetic screen, is also crucial for HSM (Friedrich *et al*., 2021). HSFA3 and HSFA2 interact and form heteromeric (likely trimeric) complexes with other HSFs like HSFA1a, HSFA1b, HSFA1d, HSFA6b and HSFA7a. Complexes containing both HSFA2 and HSFA3 exhibit the strongest activation of HSM genes. The absence of either factor weakens this memory, with complete loss observed in double‐knockout mutants. Intriguingly, only HSFA2 is required for the enhanced re‐induction of memory genes (type II memory genes). A significant overlap has been identified in the target genes of both transcription factors, which bind to the tripartite HSE‐like motif TTCtaGAAnnTTCt (Kappel *et al*., 2023). Notably, these factors do not exclusively bind to promoter regions of genes exhibiting transcriptional memory, suggesting HSFA2 and HSFA3 may regulate a broader spectrum of genes beyond those associated with transcriptional memory, thereby contributing to a more complex and nuanced regulatory system for HSM in plants.

**Fig. 3:**
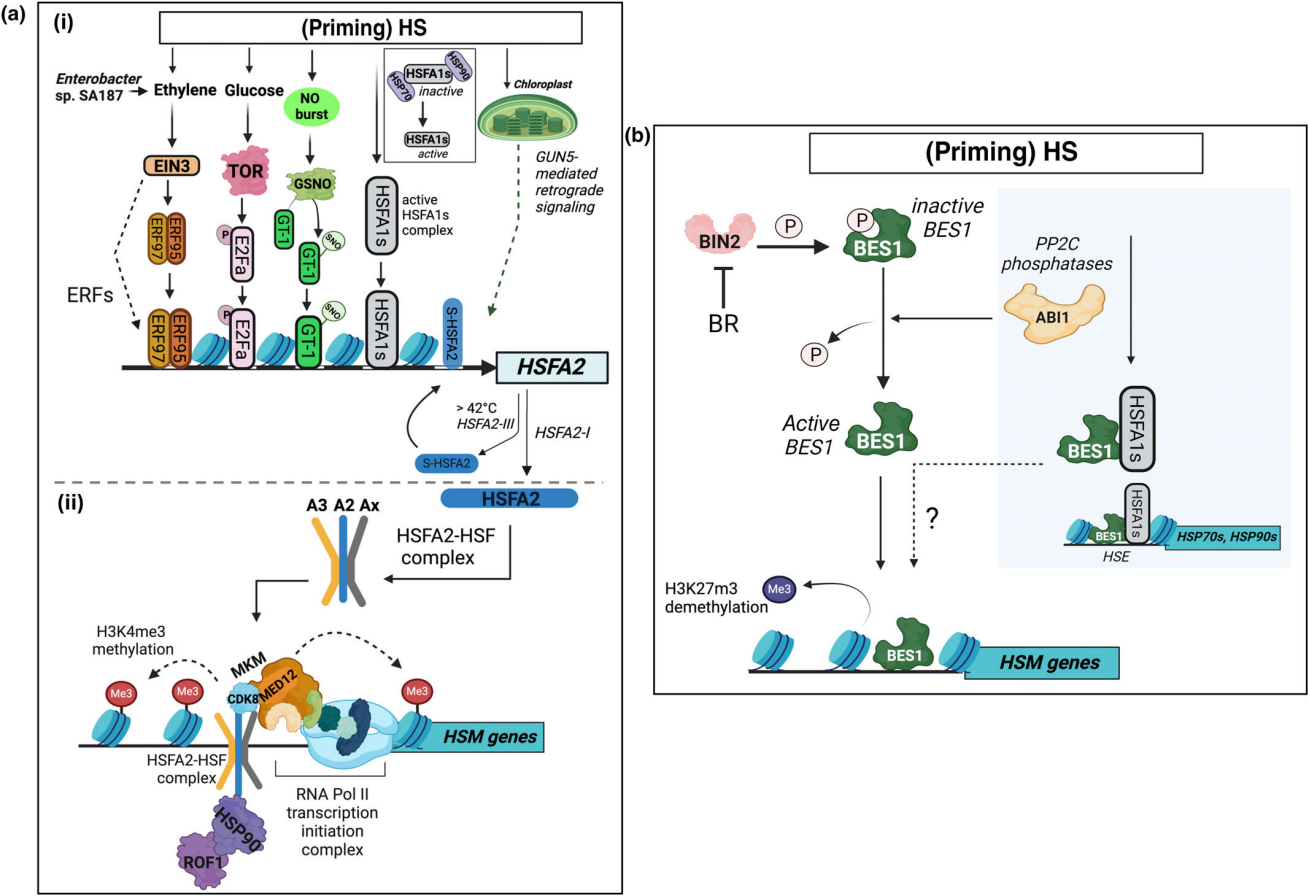
Transcriptional regulation of heat stress memory (HSM) genes upon (priming) HS. (a) HSFA2‐dependent transcriptional regulation of HSM genes in Arabidopsis. (i) Transcriptional regulation of *HSFA2*: (priming) HS and other environmental factors, such as *Enterobacter* sp. SA187 trigger different signaling cascades involving ETHYLENE INSENSITIVE3 (EIN3), TARGET OF RAPAMYCIN (TOR) or nitric oxide (NO)–S‐nitrosoglutathione (GSNO) to activate *HSFA2* transcription. This involves transcription factors like ERF95/97, E2Fa which is phosphorylated (‘P’) by TOR, and GT‐1, which is activated by S‐nitrosylation (‘SNO’). Furthermore, retrograde signaling via GUN5 plays a role in regulating *HSFA2* expression in response to HS. However, the precise molecular details of how GUN5 influences *HSFA2* transcription through retrograde signaling remain to be fully identified. (ii) Regulation of HSM genes by HSFA2: The HSFA2‐HSF complex binds to target HSM gene promoters. At these promoters, chromatin modifications, such as H3K4me3 methylation occur, which mark the genes for active transcription. HSFA2 directly binds to CDK8 and recruits the mediator complex to HSM genes, where it coordinates the activity of RNA polymerase II. Proteins like HSP90 and ROF1 form a complex with HSFA2 and enhance its transcriptional activity toward target genes. ‘A2’, ‘A3’ and ‘Ax’ indicate HSFA2, HSFA3 and other HSFs participating in complex formation. (b) Regulation of HSM genes by BES1 in Arabidopsis. BR‐induced inhibition of BIN2 kinase and (priming) HS trigger the dephosphorylation of BES1 and increase its nuclear abundance. Activated BES1 binds to the promoters of HSM genes, promoting their sustained activation. This regulatory process involves the demethylation of H3K27me3. Additionally, HS activates BES1 through ABA‐repressed PP2C phosphatases, such as ABI1. Activated BES1 then interacts with HSFA1s, binding to *HEAT SHOCK ELEMENTS* (*HSEs*) within the promoters of *HSP70* and *HSP90* genes, which induces their expression and enhances HS resistance. It remains to be determined whether the BES1‐HSFA1s complex also binds HSM genes highlighted by a question mark. Dotted lines depict processes or interactions for which the exact mechanisms are not fully elucidated. ABA, abscisic acid; ABI1, ABA INSENSITIVE 1; BES1, BRI1 EMS‐SUPPRESSOR 1; BIN2, BRASSINOSTEROID‐INSENSITIVE 2; BR, brassinosteroids; CDK8, CYCLIN‐DEPENDENT KINASE 8; ERFs, ethylene response factors; ERF95, Ethylene Response Factor 95; ERF97, Ethylene Response Factor 97; E2Fa, transcription factor E2Fa; GT‐1, trihelix transcription factor GT‐1; GUN5, GENOMES UNCOUPLED 5; HS, heat stress; HSF, heat shock factor; HSFA1s, Heat Shock Factor A1s; *HSFA2, HEAT SHOCK FACTOR A2; HSFA2‐I, HSFA2* splice variant I, encoding the full‐length protein; *HSFA2‐III, HSFA2* splice variant III, encoding the truncated protein S‐HSFA2; HSP70, Heat Shock Protein 70; HSP90, Heat Shock Protein 90; H3K4me3, histone H3 ‐ lysine 4 trimethylated; H3K27me3, histone 4 ‐ lysine 27 trimethylated; Med12, MEDIATOR 12; MKM, mediator kinase module; PP2C, protein phosphatase type 2C; RNA Pol II, RNA polymerase II; ROF1, ROTAMASE 1; S‐HSFA2, truncated HSFA2 protein. Figure assembled from part figures created in BioRender. Bz, S. (2025) https://BioRender.com/a09a574, Bz, S. (2025) https://BioRender.com/s35t757, and Bz, S. (2025) https://BioRender.com/a23i343.

*HSFA2* expression is regulated by various transcription factors (Fig. 3). The most well‐studied transcriptional regulators of the HS response, HSFA1s (a, b, d and e), are known to bind the *HSFA2* promoter (Nishizawa‐Yokoi *et al*., 2011; Yoshida *et al*., 2011). HSFA1s exhibit partial redundancy in HSM. Triple knockout mutants *hsfa1a*/*b*/*e* and *hsfa1b*/*d*/*e* resemble WT plants, whereas *hsfa1a*/*d*/*e* and *hsfa1a*/*b*/*d* mutants show reduced and abolished HSM, respectively (Liu *et al*., 2011). Expression of dominant negative versions of HSFA1s in transgenic plants (due to SRDX fusions) has revealed that only HSFA1d and HSFA1e significantly reduce *HSFA2* expression, suggesting they act as primary drivers of *HSFA2* expression under HS (Nishizawa‐Yokoi *et al*., 2011). Moreover, the trihelix transcription factor GT‐1 acts as a NO‐controlled activator of *HSFA2* (He *et al*., 2022) (see Section VI). Furthermore, heat‐induced expression of *HSFA2* is regulated by ethylene, which activates the ETHYLENE INSENSITIVE3 (EIN3) transcription factor and subsequently promotes the expression of *ETHYLENE RESPONSE FACTORS 95* and *97* (*ERF95* and *97*) (Huang *et al*., 2021). While the heat response in single and double mutants of *ERF95* and *ERF97* was comparable to that of the WT, the quadruple knockout mutant *erf95*/*96*/*97*/*98* exhibited increased sensitivity to HS. The study focused on the role of ethylene and its downstream effects on basal thermotolerance. Whether ERF95 and ERF97 might also be involved in establishing HSM warrants further investigation.

The heat‐induced expression of *HSFA2* is also impacted by a retrograde signal derived from chloroplasts, mediated by GENOMES UNCOUPLED 5 (GUN5). This regulation is important for increasing the pool of HSP21 (a transcriptional target of HSFA2) in chloroplasts and protecting Photosystem II (PSII) from damage under HS (Chen *et al*., 2017).

HSFA2‐independent regulation of HSM involves complex signaling of phytohormones, including brassinosteroids (BR) and ABA (Fig. 3). Heat‐induced nuclear accumulation of dephosphorylated BRI1 EMS‐SUPPRESSOR 1 (BES1), a key component of BR signaling, is crucial for maintaining memory gene expression by affecting histone H3K27me3 demethylation (Yao *et al*., 2022). While BR‐induced dephosphorylation of BES1 enhances HSM in Arabidopsis seedlings, BES1 can also be activated and regulated independently of BR during HS through ABA‐repressed type 2C phosphatases (PP2Cs) (Albertos *et al*., 2022). Activated BES1 interacts with HSFA1s which bind to *HEAT SHOCK ELEMENTS* (*HSEs*) in *HSP70* and *HSP90* promoters; this induces their expression and improves HS resistance. It is yet to be determined whether the BES1‐HSFA1s complex also binds to HSM genes. Future work could investigate how ABA and other phytohormones regulate and fine‐tune HSM.

Regulation of HSM also occurs at the post‐transcriptional level. Evidence suggests that ARGONAUTE1 (AGO1) and miRNA‐dependent gene silencing are involved in this process. Weak alleles of *AGO1* (*ago1‐25* and *ago1‐27*) show impaired HSM. Additionally, *miRNA156*, which binds AGO1, is crucial for suppressing *SQUAMOSA‐PROMOTER BINDING‐LIKE 2* and *11* (*SPL2* and *11*), thereby mitigating the negative effect of SPL2 on memory gene expression during later stages of HSM (Stief *et al*., 2014). Alternative splicing (AS) further contributes to HSM by increasing transcriptome and proteome diversity. Arabidopsis seedlings directly exposed to severe HS exhibit significant splicing repression through intron retention (IR) in multiple genes, including *HSFs* (*HSFA2*, *HSFA7a* and *HSFA7b*), *HSPs* (*HSP21*, *HSP70.8* and *HSP90.1*) and *HSA32*. However, plants pre‐exposed to a priming HS recover their splicing efficiency more quickly after severe HS than nonprimed plants (Ling *et al*., 2018). This priming‐induced splicing memory likely improves the plant's resilience to repeated HS and its ability to recover and resume normal growth by modulating the production of functionally relevant protein isoforms. Temperature‐dependent AS is a common trait across various plant species (Jiang *et al*., 2017; Lee *et al*., 2018; Ling *et al*., 2018; Hu *et al*., 2020; Zhang *et al*., 2020). In Arabidopsis, AS of *HSFA2* fine‐tunes its expression and protein levels. Under mild HS (37°C), a 31‐bp mini‐exon from the conserved intron in the DNA‐binding domain is included in the transcript, resulting in a *HSFA2‐II* splice variant containing a premature stop codon, which triggers degradation via nonsense‐mediated mRNA decay (NMD) (Sugio *et al*., 2009; Liu *et al*., 2013). However, exposure to severe heat (> 42°C) produces a new splice variant, *HSFA2‐III*, through a 5′ splice site within the intron. This variant results in a truncated HSFA2 protein (S‐HSFA2) that localizes to the nucleus and binds to its own promoter, leading to self‐activation of its expression (Liu *et al*., 2013). S‐HSFA2 also plays a role in the noncanonical HSR by alleviating the hyperactivation of the canonical HSR mediated by the HSFA2‐*HSP17.6B* module. Two possible mechanisms for S‐HSFA2 functions include: (1) binding to a newly identified heat‐regulated element (HRE) in the *HSP17.6B* promoter to reduce its expression and (2) interacting with the DNA‐binding domain of full‐length HSFA2 to prevent its binding to the *HSE* and inhibit *HSP17.6B* activation (Chen *et al*., 2024).

### 2. Epigenetic regulation

Epigenetic HSM has been extensively studied and reviewed in recent years (Perrella *et al*., 2022; Ramakrishnan *et al*., 2022; Nishio *et al*., 2024; Pratx *et al*., 2024). Therefore, we only briefly highlight the main drivers in the process here.

Chromatin alterations at specific genomic regions are crucial for effectively modifying transcription following heat exposure. A forward genetic screen identified *FORGETTER1* (*FGT1*), an *Arabidopsis thaliana* orthologue of *Strawberry Notch*, which functions as a transcriptional co‐activator and binds to the proximal regions of HSM genes (Brzezinka *et al*., 2016). In interactions with chromatin remodeling complexes, including Imitation SWItch (ISWI, with its components CHR11 and CHR17) and SWI/SNF (with its component BRAHMA), FGT1 changes nucleosome dynamics. This modulation helps maintain low nucleosome occupancy and ensures sustained expression of target memory genes. Another example of chromatin reorganization in response to HS is from (N. Wang *et al*., 2023). They showed that a diurnal repetitive heat regime of 37°C over 3 d affects the nuclear lamina, a mesh of proteins like lamins beneath the inner nuclear membrane. Under optimal conditions, the nuclear lamina‐associated proteins CROWDED NUCLEI 1 and 4 (CRWN1 and 4) localize at the periphery, forming plant nuclear lamina‐associated domains (PLADs), that is regions of transcriptionally less active chromatin. After HS, CRWN1 relocates toward the center of the nucleus, leading to chromatin reorganization, partial dissociation from PLADs and activation of genes like *HSP17.4*, *HSP22.0* and *HSA32* in those regions. Although this study showed dynamic changes in the nuclear structural components and their impact on gene expression in response to temperature variation, it is important to investigate whether this mechanism also assists in maintaining gene expression and establishing HSM. A recent study in tomato (*Solanum lycopersicum*) demonstrated the effect of HS on changes in the 3D chromatin structure to regulate gene expression (Huang *et al*., 2023). HS transiently opened chromatin regions with proximal regulatory elements (RE) enriched with histones presenting specific epigenetic marks, that is H3K9ac, H3K18ac, H3K27ac and H3K4me3, while distal REs are marked with nucleosomes rich in H3K9ac and H3K18ac, but limited in H3K4me3. These newly accessible RE were bound by SlHSFA1a, the central transcriptional regulator of the HS response. SlHSFA1a also bound to promoters of HSR genes, for example *Solyc09g074475* and *HSFA2*, forming chromatin loops between enhancer and promoter regions to dynamically regulate gene expression in response to heat.

Epigenetic memory is also established through trimethylation of lysine 4 on histone H3 (H3K4me3). The process is facilitated by the methyltransferases ARABIDOPSIS TRITHORAX 1 (ATX1) and SET DOMAIN GROUP 25 (SDG25). The *atx1sdg25* double‐knockout mutant shows decreased H3K4me3 levels and expression of several HSR genes during the recovery from HS. However, HSM remains unaffected in the double mutant, suggesting the involvement of other regulatory layers crucial for maintaining it (Song *et al*., 2021).

CYCLIN‐DEPENDENT KINASE 8 (CDK8) is another essential component for accumulating H3K4me3 methylation at type II HSM genes. Together with MEDIATOR 12 (MED12), a part of the kinase module within the Mediator complex, CDK8 participates in regulating HSM genes and facilitates the re‐induction of gene expression (Crawford *et al*., 2024). The binding of an HSFA2/HSFA3 heteromeric complex to the promoters of HSM genes promotes sustained accumulation of H3K4me3 (Friedrich *et al*., 2021). Furthermore, HSFA2 directly binds to CDK8 and recruits the Mediator complex to HSM genes, where it coordinates the activity of RNA polymerase II (Crawford *et al*., 2024) (Fig. 3). Additionally, glucose‐induced HIKESHI‐LIKE PROTEIN1 (HLP1) is required for maintaining H3K4me3 marks at HSM genes (Sharma *et al*., 2019) (see Section VI).

Another factor essential for maintaining memory gene expression is the chromatin‐associated protein BRUSHY1 (BRU1)/TONSOKU/MGOUN3. Knocking out *BRU1* does not alter the initial induction of HS‐responsive gene expression but lowers the maintenance of memory gene transcripts, indicating that BRU1 copies chromatin marks onto newly replicated DNA (Brzezinka *et al*., 2019; Perrella *et al*., 2022). In addition to the favorable H3K4me3 histone mark, removal of repressive histone marks is crucial for HSM. JUMONJI demethylases (including JMJ11, JMJ12, JMJ30 and JMJ32) have been shown to remove H3K27me3 modification at *HSP22* and *HSP17.6C* genes, preparing them for subsequent activation (Yamaguchi *et al*., 2021).

### 3. Proteome integrity

Protein adaption is essential for preserving cellular homeostasis and mitigating damage caused by elevated temperatures. Molecular chaperones like HSPs prevent protein denaturation and potentially detrimental protein aggregation. Under naive (non‐HS) conditions, protein folding is mainly controlled by constitutively expressed members of the *HSP60*, *HSP70* and *HSP90* families. During HS, additional inducible HSPs, including small HSPs, are expressed to prevent aggregation (Fragkostefanakis *et al*., 2015). Findings indicate that sustained levels of stress‐related proteins, primarily HSPs, are important for HSM. HSP21 (also known as HSP25.3‐P), a nuclear‐encoded small plastidial protein, is strongly expressed after heat priming and is regulated by HSFA2 and HSFA3 (Zhong *et al*., 2013; Kappel *et al*., 2023). Knocking down *HSP21* impairs priming‐induced HS tolerance in transgenic plants, while its overexpression enhances HSM (Sedaghatmehr *et al*., 2016). In accordance, sustained high levels of HSP21 during the recovery phase are essential for effective HSM in natural accessions of Arabidopsis. The precise role of HSP21 in HSM is not fully understood, but insights from experiments on basal thermotolerance may provide clues. HSP21 functions downstream of GUN5‐mediated retrograde signaling during HS (Chen *et al*., 2017). Heat‐induced activation of HSFA2 and its target genes, including *HSP21*, is significantly reduced in the *gun5* mutant. Notably, overexpression of *HSP21* restores heat sensitivity in *gun5* by stabilizing PSII and maintaining thylakoid membrane integrity. HSP21 directly interacts with PSII proteins D1 and D2, protecting them from heat‐induced damage (Chen *et al*., 2017). Additionally, HSP21 forms a complex with the plastid nucleoid protein pTAC5 and, together with plastid‐encoded RNA polymerase (PEP), supports chloroplast development under HS conditions (Zhong *et al*., 2013). Structurally, HSP21 assembles into dodecameric complexes crucial for chaperone activity (Rutsdottir *et al*., 2017). Another small HS‐related protein important for HSM is HSA32. Its expression rapidly increases within 2 h after HS then declines during recovery, but the protein accumulates later, suggesting a role in long‐term adaptation rather than immediate thermotolerance (Charng *et al*., 2006). The interplay between HSA32 and HSP101 establishes a positive feedback loop for regulating HSM and prolonged heat acclimation. HSP101 is a chaperone that solubilizes aggregated proteins during severe HS (Wu *et al*., 2013). Although HSP101 does not exhibit transcriptional memory, its sustained presence is crucial for maintaining high levels of HSA32 and extending HSM. In turn, HSA32 stabilizes HSP101 by inhibiting its degradation, reinforcing levels of both proteins (Wu *et al*., 2013). The interaction between HSA32 and HSP101 is conserved between species (Lin *et al*., 2014). *HSA32* orthologues are found across a wide range of land plants, including crops like tomato and rice, highlighting its evolutionary conservation and fundamental role in the HSR across species (Liu *et al*., 2006; Lin *et al*., 2014). HSP90.1 also plays a significant role in HSM. Among the four homologous cytosolic *HSP90* genes in Arabidopsis, *HSP90.1* is weakly expressed under normal conditions but induced by heat (Yamada *et al*., 2007). HSP90.1 interacts with the peptidyl prolyl *cis*‐*trans* isomerase protein ROTAMASE 1 (ROF1), a homolog of mammalian FKBP4/FKBP52, to form a cytoplasmic complex under nonstress conditions. Upon heat exposure, HSP90.1 binds to HSFA2, and the HSFA2‐HSP90.1‐ROF1 complex moves to the nucleus, where it enhances the transcriptional activity of HSFA2 on its target genes (Aviezer‐Hagai *et al*., 2007; Meiri & Breiman, 2009). Higher accumulation of both ROF1 and HSP90.1 during the recovery phase (by blocking their autophagy‐mediated degradation; see Section V) enhances plant HSM capacity (Thirumalaikumar *et al*., 2021). The examples above underscore the importance of stress protein stability for HSM. Nevertheless, the mechanisms of many priming‐induced proteins remain unclear. Exploring other protein families beyond chaperones could provide valuable insights into broader mechanisms of HSM and plant resilience.

### 4. Metabolic regulation

To adapt to fluctuating temperatures, plants rapidly alter their metabolic composition to maintain homeostasis. After heat treatment, Ca^2+^ ions accumulate inside cells and bind CALMODULIN 3 (CaM3), which interacts with CaM‐BINDING PROTEIN KINASE 3 (CBK3). CBK3 phosphorylates HSFA1a, and together with CaM3, is crucial for HS signal transduction (Liu *et al*., 2008). Intracellular Ca^2+^ levels increase through external influx and release from internal stores via the phosphatidylinositol pathway (Finka *et al*., 2012; Gao *et al*., 2012). In Arabidopsis, PHOSPHOLIPASE C 9 (PLC9), a membrane‐bound enzyme, converts phosphatidylinositol 4,5‐ bisphosphate (PIP_2_) to inositol 1,4,5‐triphosphate (IP_3_), thereby elevating intracellular Ca^2+^ levels during a moderate heat exposure (37°C) (Zheng *et al*., 2012). *Plc9* knockouts show reduced basal and acquired thermotolerance, but PLC9 involvement in HSM has yet to be determined. Additionally, PHOSPHOLIPASE D ALPHA 2 (PLDα2) hydrolyzes membrane lipids into phosphatic acid (PA), acting as a signaling molecule and modulating HSM (Urrea Castellanos *et al*., 2020). PLDα2 is directed to plasma membranes by FORGETTER2 (FGT2, APD9/P2C79), a type 2C protein phosphatase that may regulate PLDα2 phosphorylation.

A comprehensive metabolic analysis of Arabidopsis identified metabolites exhibiting memory patterns, some of which remain elevated for up to 4 d (Serrano *et al*., 2019). Notably, raffinose family oligosaccharides (RFOs), branched‐chain amino acids (BCAAs) and tocopherols were enriched, suggesting roles as osmolytes, antioxidants and growth precursors. RFOs like galactinol and raffinose are particularly interesting owing to their biosynthesis being regulated by HSFA2 and their potential role in ROS scavenging during HS (Nishizawa *et al*., 2008). By contrast, nonprimed plants accumulate metabolites like amino acids, RNA catabolites and lysolipids, indicating they experience greater heat effects than primed plants. Twenty‐one metabolites increased more steeply in primed plants under recurring HS, indicating a more efficient metabolic response.

Another study revealed a HS‐dependent accumulation of extra‐chloroplastic polyunsaturated triacylglycerols (TGs) during HS (32–50°C), which was reversible after returning plants to normal growth temperature. Although TGs may support membrane remodeling during initial heat acclimation, a role in HSM is perhaps unlikely. Notably, TG accumulation does not depend on the HSFA1 master regulators or HSFA2 (Mueller *et al*., 2015).

Glucose is crucial for maintaining HSM by regulating the plant's priming response. After severe HS, glucose accumulation is higher in previously primed plants (Olas *et al*., 2021). While glucose starvation may not affect HS‐induced gene transcripts shortly after HS, it disrupts the type I memory expression pattern of HSM genes, highlighting the importance of sugar supply by glucose in sustaining HSM (Sharma *et al*., 2019).

A role of ethylene signaling in activating HSM genes was demonstrated by employing the endophytic bacterium *Enterobacter* sp. SA187, isolated from the desert shrub *Indigofera argentea*, which enhances plant thermotolerance (Andrés‐Barrao *et al*., 2017; de Zélicourt *et al*., 2018; Shekhawat *et al*., 2021). SA187 promotes HSFA2‐dependent H3K4me3 marks at memory gene loci, for example *ASCORBATE PEROXIDASE 2* (*APX2*) and *HSP18.2*, even under naive conditions, mimicking thermopriming‐like features (Shekhawat *et al*., 2021). Unlike thermopriming, which activates *HSFA2* through HSFA1s, SA187 regulates *HSFA2* via the ETHYLENE‐INSENSITIVE 3 (EIN3) transcription factor. Additionally, the histone marks induced by SA187 are constitutively present, facilitating the induction of gene expression throughout plant development. Furthermore, John *et al*. (2024) showed that HSFA7b directly binds to the *EIN3* promoter to activate the transcriptional HS cascade and establish HSM.

### 5. Biomolecular condensates and HSM

Biomolecular condensates are membrane‐less organelles typically formed by liquid–liquid phase separation (LLPS). These structures concentrate proteins, nucleic acids and metabolites within cells and are found in various compartments, for example cytosol and nucleus. Conserved across eukaryotes, condensates are crucial for numerous biological processes. In plants, recent evidence suggests that condensates play essential roles in development and responses to environmental change (Londoño Vélez *et al*., 2022; Field *et al*., 2023; Q. Liu *et al*., 2024). Additionally, the discovery that the thermosensory protein ELF3 forms nuclear condensates (speckles) in Arabidopsis, regulating short‐term memory of daytime temperatures, suggests their potential role in memory processes (Murcia *et al*., 2022). However, whether condensates are involved in other types of memory, such as HSM, remains unknown. In response to HS, cells transiently form stress granules (SGs) to mitigate stress effects. In Arabidopsis, two recently identified *glycine‐rich RNA‐binding proteins*, RBGD2 and RBGD4, undergo LLPS *in vitro*, and their condensation into SGs is crucial for enhancing heat tolerance (Zhu *et al*., 2022). Whether they also contribute to HSM is yet to be determined. Interestingly, some components necessary for HSM are associated with SGs and protected during HS. *HSFA7b* and *HSFA2* mRNAs are recruited into SGs by RNA‐binding acetylation lower binding affinity (ALBA) proteins (Tong *et al*., 2022), whereas AGO1, a protein involved in RNA silencing, undergoes LLPS and condenses into SGs through its prion‐like domain (Blagojevic *et al*., 2024). This sequestration may influence the availability, stability and activity of these components, potentially affecting their role in stress memory. However, as ALBA mutants do not show impaired acquired thermotolerance (Tong *et al*., 2022), the significance of SGs in HSM remains uncertain, highlighting the need for further investigation.

Studies in yeast and Drosophila suggest that the temporal and spatial condensation of proteins involved in cellular decision‐making can contribute to a memory of past experiences (Reichert & Caudron, 2021), for example spatial reorganization of the cyclin‐dependent kinase inhibitor Far1, which transmits a memory of past pheromone exposure across generations in yeast (Doncic *et al*., 2015). In the nucleus, condensates may concentrate epigenetic modifiers at specific loci and compartmentalize transcription factors and co‐regulators, facilitating their interaction with target genes (Pirrotta & Li, 2012; Chowdhary *et al*., 2022; Zhang *et al*., 2022). The thermally induced relocation of the thermosensory protein TWA1 into specific nuclear subdomains, where it forms a repressor complex to activate *HSFA2* and *HSP21* expression, could potentially link it to HSM due to the involvement of these genes in memory processes (Bohn *et al*., 2024).

Long‐term storage and controlled release of biomolecules could also serve as mechanisms for memory and adaptation. In *Saccharomyces cerevisiae*, this is exemplified by inhibition of PKA, an evolutionarily conserved protein kinase A that regulates formation of ribonucleoprotein granules. These granules store and repress certain transcripts, allowing their later gradual release and translation, facilitating long‐term memory and adaptation to subsequent stress (Jiang *et al*., 2020). However, analogous research in plants is lacking. Also, most studies have focused on condensate formation immediately after heat shock rather than during the recovery phase. These recovery‐phase condensates could potentially serve as crucial storage sites or specialized microenvironments for regulating stress memory or resetting.

## V. Mechanisms of HS resetting

**Fig. 4:**
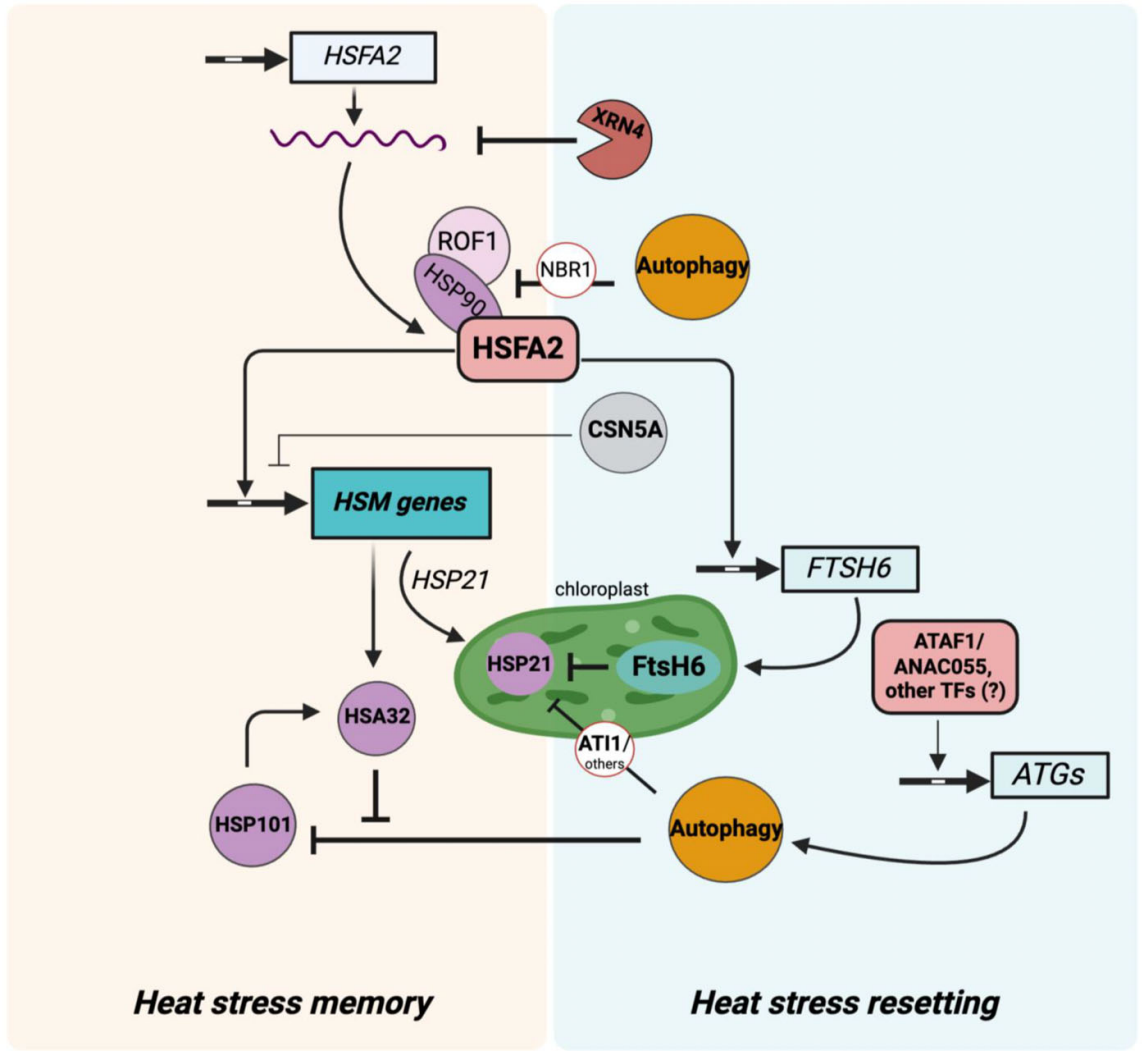
Simplified model summarizing mechanisms of heat stress (HS) resetting and their connection to HS memory (HSM). In Arabidopsis, HSFA2 activates memory genes (e.g. *HSA32* and *HSP21*) that promote and maintain HSM, and activates the memory gene involved in resetting, *FTSH6*. During recovery, the resetting of *HSFA2* transcripts involves mechanisms like mRNA decay via XRN4. Additionally, the COP9 signalosome subunit CSN5A aids in transcriptional resetting by downregulating HSM gene expression (e.g. *APX2* and *HSP22*; not specified in the figure), potentially through altered H3K4me3 methylation, a process that requires further investigation. Protein degradation is also crucial: the plastidial metalloprotease FtsH6 degrades HSP21 to limit memory duration. Autophagy, mediated by NBR1 and ATI1 receptors, target key chaperones like HSP101, HSP90‐ROF1 and HSP21. The degradation of HSP90‐ROF1 impacts HSFA2 activity and its transcriptional regulation of target genes. Transcription factors ATAF1 and ANAC055, which are linked to autophagy regulation, are involved in stress resetting, as their mutants show improved memory retention. This network of transcriptional and post‐translational mechanisms suggests how plants balance HSM and resetting for both effective stress response and resumption of growth. Arrow‐ending lines indicate positive, and T‐ending lines negative interactions, respectively. ANAC055, Arabidopsis NAC (NAM, ATAF and CUC) transcription factor 055; APX2, ASCORBATE PEROXIDASE 2; ATAF1, Arabidopsis NAC (NAM, ATAF and CUC) domain containing protein ATAF1; *ATGs, AUTOPHAGY RELATED GENES*; ATI1, ATG8‐INTERACTING PROTEIN 1; CSN5A, CONSTITUTIVE PHOTOMORPHOGENESIS 5A; FtsH6, Filamentous Temperature‐Sensitive H6 protein; *FTSH6, FILAMENTOUS TEMPERATURE‐SENSITIVE H6* gene; HSA32, Heat Stress‐Associated 32 kDa Protein; HSFA2, Heat Shock Factor A2; HSP21, Heat Shock Protein 21; HSP22, Heat Shock Protein 22; HSP90, Heat Shock Protein 90; HSP101, Heat Shock Protein 101; H3K4me3, histone H3 ‐ lysine 4 trimethylation; NBR1, NEXT‐TO‐BRCA1; ROF1, ROTAMASE 1; TFs, transcription factors; XRN4, 5'‐3' EXORIBONUCLEASE 4. Created in BioRender. Bz, S. (2025) https://BioRender.com/g63v207.

The capacity of plants to timely recover after a stress experience is key to reaching a new state of homeostasis, allowing optimal growth and development once the stress has subsided. While stress memory has received considerable attention in recent years, few studies have examined how plants recover when favorable conditions return. A review by Crisp *et al*. (2016) conceptualizes the importance of recovery from stress and discusses mechanisms for resetting at the epigenome and transcriptome levels. Here, we discuss recent advancements in understanding recovery from HS and resetting of HSM (Fig. 4). Several studies have shown a rapid reversal of most changes in the transcriptome following recovery from HS (Stief *et al*., 2014; Liu *et al*., 2018; Friedrich *et al*., 2021; Alshareef *et al*., 2022). Friedrich *et al*. (2021) examined the transcriptomic responses of Arabidopsis seedlings using RNA‐seq shortly after heat priming (4 h) and during subsequent recovery periods (28 h and 52 h). In Col‐0 seedlings, 3381 genes were activated in response to heat priming, of which most reverted to their baseline levels within 28 h postpriming. However, a small fraction (156 genes, < 5%) exhibited a type I memory pattern, showing continued elevated expression at the 28‐h and 52‐h time points. Notably, 74.4% of these genes did not maintain high expression at 52 h in the *hsfa2hsfa3–1* double mutant, highlighting the critical roles of both transcription factors in sustaining heat‐induced gene expression.

Few studies have explored the dynamics of HS (memory) gene expression over prolonged recovery periods and the resetting mechanisms responsible. While attenuation of upstream transcription factors is an important mechanism behind transcriptional resetting, other possibilities have been suggested, for example targeted decay of transcripts or alterations in epigenetic marks. Arabidopsis 5′‐3′ EXORIBONUCLEASE 4 (AtXRN4), involved in mRNA decay, accelerates the reversion of *HSFA2* and its target (*HSP70*) transcripts to their prestress expression state after HS and during recovery. Thus, lack of XRN4 causes enhanced heat tolerance in heat‐acclimated plants (Nguyen *et al*., 2015). Targeted transcript degradation during HS recovery is also demonstrated by the *miR156‐SPL* module. Heat‐induced *miR156* specifically targets and downregulates *SQUAMOSA‐PROMOTER BINDING PROTEIN‐LIKE* transcription factors (*SPL2* and *SPL11*), preventing suppression of HSM‐related genes during recovery (Stief *et al*., 2014). CONSTITUTIVE PHOTOMORPHOGENESIS 5A (CSN5A), a subunit of the COP9 signalosome, plays a role in resetting the transcriptional memory of *APX2* and *HSP22* (Singh *et al*., 2021). Expression of the two memory genes increased 3 h after heat treatment in both Col‐0 and *csn5a‐1* hypomorphic mutants. However, whereas expression in Col‐0 returned to baseline levels within 3 d, it remained elevated in *csn5a‐1*. Further work is needed to determine the specific role of CSN5A in regulating transcriptional memory and whether it extends to other memory genes. Although H3K4me3 methylation levels at the *APX2* and *HSP22* loci were higher in *csn5a‐1* than Col‐0, suggesting a potential mechanism, methylation enrichment was also observed in the intergenic regions of these genes and at the locus of *HSP70*, where expression was high but not differentially expressed between Col‐0 and *csn5a‐1*. Lämke *et al*. (2016) demonstrated that sustained induction of HSM genes through H3K4 methylation requires functional HSFA2. Therefore, it may be interesting to investigate whether CSN5A and HSFA2 interact to regulate H3K4 methylation levels.

While most studies have focused on the transcriptional regulation of memory, research on translational control and post‐translational modifications of HSM remains limited. Various factors can impede, delay or enhance the translation of existing mRNAs. For example, in Arabidopsis and crops, studies have documented alterations in mRNA translation efficiency and selective association of stress‐induced mRNAs with polysomes in response to different stressors (Branco‐Price *et al*., 2008; Yángüez *et al*., 2013; Li *et al*., 2018; Son & Park, 2023). Furthermore, studies in yeast and plants show that mRNAs can undergo degradation while they are associated with translating ribosomes through a process called mRNA co‐translational decay (Hu *et al*., 2009; Pelechano *et al*., 2015; Guo *et al*., 2023). Recently, Dannfald *et al*. (2024) analyzed polysome‐related processes by comparing plants primed with mild heat before HS to nonprimed plants. A significant decrease in the translational efficiency of nonstress‐related mRNAs was observed, suggesting that their downregulation after stress occurs at the translational level. Priming also markedly enhanced the overall recovery of plant translation potential soon after HS, which might be essential for resuming normal growth. Further research employing experimental setups to examine HS memory is required to enhance our understanding of the impacts of transcriptional responses on establishing or resetting HSM.

Increasing evidence suggests that regulation of *in vivo* protein stability is an important mechanism for HS resetting. The regulated degradation of stress‐responsive proteins and their recycling during recovery may be an energy‐saving strategy to restore growth between stress episodes. Sedaghatmehr *et al*. (2016) showed that degradation of the small plastidial HSP21 protein by the plastid‐localized metalloprotease FtsH6 shortens maintenance of the primed state and the HSM phase duration. The role of the plastidial FtsH6‐HSP21 regulatory module in HSM was demonstrated by genetic variation in natural accessions of Arabidopsis with differing HSM capacities. *FTSH6* is a type I memory gene directly regulated by HSFA2 (Sedaghatmehr *et al*., 2022) and HSFA3 (data extracted from Kappel *et al*., 2023). By degrading HSP21, FtsH6 reduces HSM and, therefore, acts as an element of a negative feedback mechanism. This example illustrates that not all memory genes necessarily promote memory; some are integral to resetting processes to achieve homeostasis, which is crucial for a balanced stress response and growth.

Additionally, autophagy, an evolutionarily conserved pathway for degradation and recycling of damaged or unwanted substrates, serves as a resetting mechanism to limit the duration of HSM (Sedaghatmehr & Balazadeh, 2024). Autophagy is promptly induced after HS in Arabidopsis and other plant species (Zhou *et al*., 2013, 2014; Zhai *et al*., 2016; Huo *et al*., 2020) and is crucial for degrading and recycling proteins damaged by stress exposure. Plants lacking effective autophagy exhibit increased sensitivity to acute HS events. Interestingly, the function of autophagy in regulating HSM varies. During moderate HS, typically used in priming experiments, autophagy is activated and remains active throughout the recovery phase. As recovery progresses, autophagy selectively targets and breaks down various HSPs essential for memory retention. In cases of autophagy deficiency, these HSPs accumulate and enhance the plant's capacity for HS memory (Sedaghatmehr *et al*., 2019).

One of the cytosolic HSPs selectively degraded by autophagy is HSP101. HSP101 does not follow a transcriptional memory pattern, but its sustained accumulation, regulated by HSA32, is crucial for maintaining thermomemory (Wu *et al*., 2013). Thus, HSFA32 aids ‘remembering’ by stabilizing and promoting the accumulation of HSP101, whereas autophagy assists ‘forgetting’ by degrading HSP101 once it is no longer needed. The interplay between protein stabilization and degradation highlights the complex mechanisms of cellular memory and resetting. An optimal balance between these processes is essential to allow cells to adapt to and recover from repeated stress exposure.

Thirumalaikumar *et al*. (2021) demonstrated that HSP90.1 and its co‐chaperone ROF1 (AtFKBP62) are selectively degraded via autophagy, mediated by the receptor protein NBR1 (NEXT‐TO‐BRCA1), a plant homolog of the mammalian autophagic cargo receptor p62. Degradation of these proteins reduces the transcriptional activity of *HSFA2*, reducing the continuous expression of HSPs, which helps to shut down the HS response and restore cellular homeostasis. Several translation‐associated/ribosomal proteins were identified as NBR1 cargo proteins, indicating that NBR1 functions as a receptor for selective autophagy of ribosomal proteins (ribophagy) during recovery from HS. Ribophagy could affect proteome integrity, possibly through mechanisms linked to ribosome turnover or heterogeneity, marking an exciting area for future research.

Interestingly, autophagy also maintains the proteome integrity of organelles during HS recovery by complementing FtsH6 in degrading plastidial HSP21 (Sedaghatmehr *et al*., 2021). Experimental evidence suggests involvement of the cargo receptor ATG8‐INTERACTING PROTEIN1 (ATI1) in mediating HSP21 degradation via autophagy, facilitating its transfer from chloroplasts to the vacuole. Other receptors involved in this process remain to be identified. The complex, multi‐level regulatory mechanisms that prevent overaccumulation of HSP21 after stress alleviation demonstrate the sophistication and critical nature of regulating stress resetting in plants. Future research could provide deeper insights into the interplay between the autophagy and FtsH6 pathways, particularly regarding HSP21 degradation. Investigation of the activity of these pathways and degradation kinetics of HSP21 in autophagy and *ftsh6* single and double mutants or use of pharmacological pathway inhibitors could reveal how these mechanisms are coordinated. Additionally, other protein degradation pathways involved in the stress resetting process should be explored.

The regulation of autophagy following priming and during recovery remains unclear. However, two NAC transcription factors, ATAF1 and ANAC055, are promising candidates for further investigation. Both *ATAF1* and *ANAC055* are induced by priming (Alshareef *et al*., 2022; and data extracted from Friedrich *et al*., 2021). Knockout mutants of these factors exhibit improved HSM, indicating their likely involvement in stress resetting. Gene expression profiling has revealed that while priming‐induced HSPs and known memory genes are not significantly affected in *ATAF1* overexpression or knockout lines, this transcription factor appears to directly target genes involved in autophagy, as well as those related to cell wall biosynthesis and expansion (Alshareef *et al*., 2022).

## VI. Tissue‐specific responses to HSM: role of the shoot apex

**Fig. 5:**
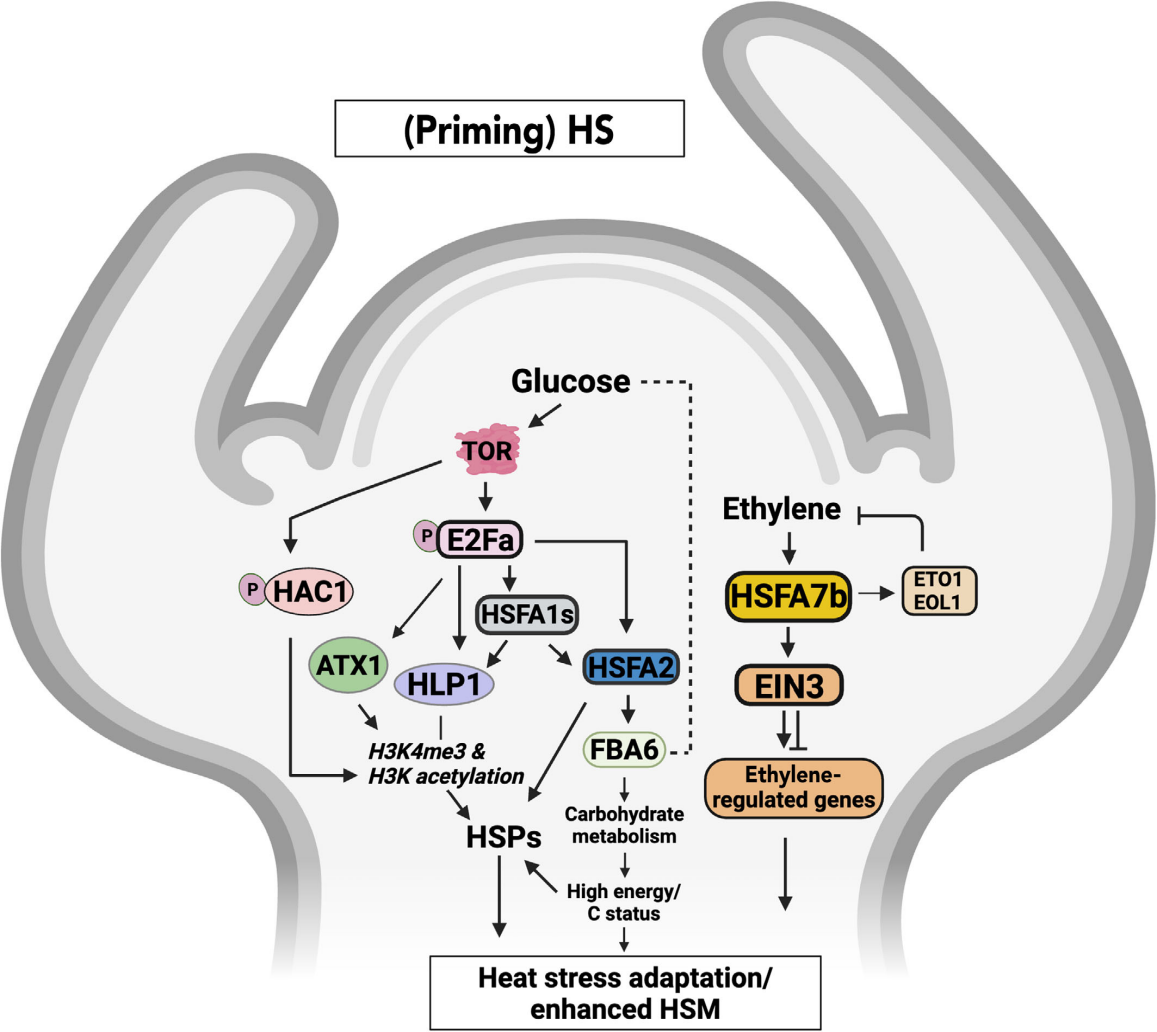
Schematic representation of signaling pathways involved in heat stress memory (HSM) at the shoot apex. Glucose signaling activates the TARGET OF RAPAMYCIN (TOR) pathway, leading to phosphorylation (‘P’) of E2Fa, which then activates HLP1 to promote histone modifications (H3K4me3 and H3K acetylations, H3K9, H3K14, H3K18, H3K23 and H3K27) at HS gene promoters in Arabidopsis. E2Fa also binds to the promoters of *HSFA1s* and *HSFA2* to activate their expression, leading to elevated expression of *HSPs* and enhanced carbohydrate metabolism through FBA6, contributing to high energy/carbon status and HSM. Glucose‐TOR‐regulated E2Fa also enhances H3K4me3 methylation at memory loci by inducing *ATX1*. Furthermore, the TOR substrate HISTONE ACETYLTRANSFERASE 1 (HAC1) regulates the expression of HS‐responsive genes by modulating acetylation at their promoter regions. Additionally, ethylene activates HSFA7b and ETHYLENE INSENSITIVE3 (EIN3). EIN3 promotes the expression of ethylene‐regulated genes, further contributing to the plant's HS adaptation. HSFA7b activates the ethylene biosynthesis repressors ETO1 and EOL1 to ensure ethylene homeostasis at the SAM. Arrow‐ending lines indicate positive, and T‐ending lines negative interactions, respectively. The dotted line indicates change in glucose availability by FBA6. ATX1, ARABIDOPSIS TRITHORAX 1; C, carbon; EOL1, ETO1‐LIKE 1; ETO1, ETHYLENE OVERPRODUCER 1; E2Fa, transcription factor E2Fa; FBA6, FRUCTOSE‐BISPHOSPHATE ALDOLASE 6; HLP1, HIKESHI‐LIKE PROTEIN 1; HS, heat stress; HSFA1s, Heat Shock Factor A1s; HSFA2, Heat Shock Factor A2; HSFA7b, Heat Shock Factor A7b; HSPs, heat shock proteins; H3K, histone 3 lysine; H3K4me3, histone H3 ‐ lysine 4 trimethylation; H3K9, H3K14, H3K18, H3K23, H3K27, histone 3, acetylated at lysine 9, 14, 18, 23, or 27, respectively; TOR, TARGET OF RAPAMYCIN. Created in BioRender. Bz, S. (2025) https://BioRender.com/p86j616.

Studies on HSM in Arabidopsis have predominantly focused on whole seedlings, often neglecting tissue‐specific responses important for comprehensively understanding plant adaptation to stress. In higher plants, the shoot apical meristem (SAM) is crucial for developing aboveground organs and plant growth. Although molecular‐regulatory mechanisms controlling the structure and maintenance of SAM under normal conditions are well understood (Dodsworth, 2009; Somssich *et al*., 2016; Demesa‐Arevalo *et al*., 2024), knowledge about how the shoot apex, and the SAM in particular, responds to and recovers from abiotic stress remains limited. Recent research has uncovered distinct responses to HS priming at the SAM (Fig. 5).

Olas *et al*. (2021) discovered that the shoot apex of Arabidopsis more rapidly responds to and recovers from HS than other plant organs. Genome‐wide transcriptional profiling revealed insights into the responsiveness at the shoot apex, suggesting a specialized mechanism for faster HSM and resetting compared with other plant parts. *HSFA2* was among the HSM genes showing a more rapid response at the shoot apex. The HSM genes include stem cell regulators *CLAVATA1* (*CLV1*) and *CLV3*, which act together with transcription factor WUSCHEL (WUS) to maintain SAM function (Somssich *et al*., 2016). Heat priming downregulated *CLV1* and *CLV3* with stronger effects after a triggering HS, but their expression fully recovered within 24 h after triggering. By contrast, unprimed plants lacked *CLV1*/*3* expression in the SAM after triggering HS (Olas *et al*., 2021). The rapid recovery in primed plants suggests that priming shields the SAM from high‐temperature stress's harmful and growth‐restricting effects.

As a stem cell center, the SAM lacks fully functional chloroplasts and relies on a carbon supply from other organs, such as cotyledons and true leaves. Interestingly, sugar metabolism genes like *FRUCTOSE‐BISPHOSPHATE ALDOLASE 6* (*FBA6*) and *PLASTIDIAL PYRUVATE KINASE 4* (*PKP4*) have been identified as HSM genes. Experimental evidence indicates that FBA6 functions as a HSM factor in the Arabidopsis SAM and is directly regulated by HSFA2 (Olas *et al*., 2021).

Additionally, glucose acts in HSM as a signaling molecule through the TARGET OF RAPAMYCIN (TOR) – E2Fa transcription factor module. Glucose and heat activate TOR signaling in the SAM, which phosphorylates and activates E2Fa to regulate HS‐induced genes, including *HSFA1s* and *HSFA2* (Sharma *et al*., 2019, 2021). E2Fa and HSFA1s induce *HIKESHI‐LIKE PROTEIN 1* (*HLP1*), essential for HS adaptation. *HLP1* knockout mutants show reduced thermotolerance, while *HLP1* overexpression confers enhanced tolerance (Sharma *et al*., 2019). HLP1 promotes acetylation of histone H3 at HS gene promoters, facilitating transcriptional activation; it is also essential for maintaining H3K4me3 marks at HSM genes. Furthermore, glucose‐TOR‐regulated E2Fa promotes H3K4me3 methylation at memory loci through ATX1 induction (Sharma *et al*., 2022). Moreover, the TOR substrate HISTONE ACETYLTRANSFERASE 1 (HAC1) coordinates HS‐responsive gene expression by regulating acetylation at their promoters (Sharma *et al*., 2021).

Rapid transcriptional activation of *HSFA2* at the shoot apex, followed by its later activation in other tissues, suggests the involvement of a mobile signal originating from the apex and triggering *HSFA2* activation in other organs. He *et al*. (2022) provided evidence that NO, or its intracellular reservoir S‐nitrosoglutathione (GSNO), serves as a mobile signaling molecule. NO levels rapidly increased after HS at the shoot apex, while HS‐induced activation of *HSFA2* was reduced in the NO‐deficient *noa1* mutant but more uniformly distributed in the NO overproducer *nox1*.

GT‐1, a trihelix transcription factor, binds to its AATTAT *cis*‐RE in the *HSFA2* promoter in an HS‐dependent manner. Mutants lacking GT‐1 show reduced HS tolerance (He *et al*., 2022), and S‐nitrosylation of GT‐1 by treatment with GSNO at low concentrations (5–50 μM) enhances binding of GT‐1 to the NO‐responsive AATTAT element. S‐nitrosylation, involving attachment of an NO group to a thiol in cysteines of proteins, forming an S‐nitrosothiol, represents an important biochemical mechanism of redox‐based signaling (Hess *et al*., 2005; Y. Liu *et al*., 2024).

Ethylene homeostasis at the SAM is also critical for HSM. John *et al*. (2024) demonstrated that impairing HSFA7b function temporarily reduces SAM activity following priming. HSFA7b directly affects ethylene responses by binding to the *EIN3* promoter, thereby activating the transcriptional HS cascade and establishing HSM. Concurrently, HSFA7b upregulates negative regulators of ethylene biosynthesis, including *ETHYLENE OVERPRODUCER 1* (*ETO1*) and *ETO1‐LIKE 1* (*EOL1*), ensuring the fine‐tuning of ethylene levels in the SAM during severe HS conditions.

## VII. Future perspectives

**Fig. 6:**
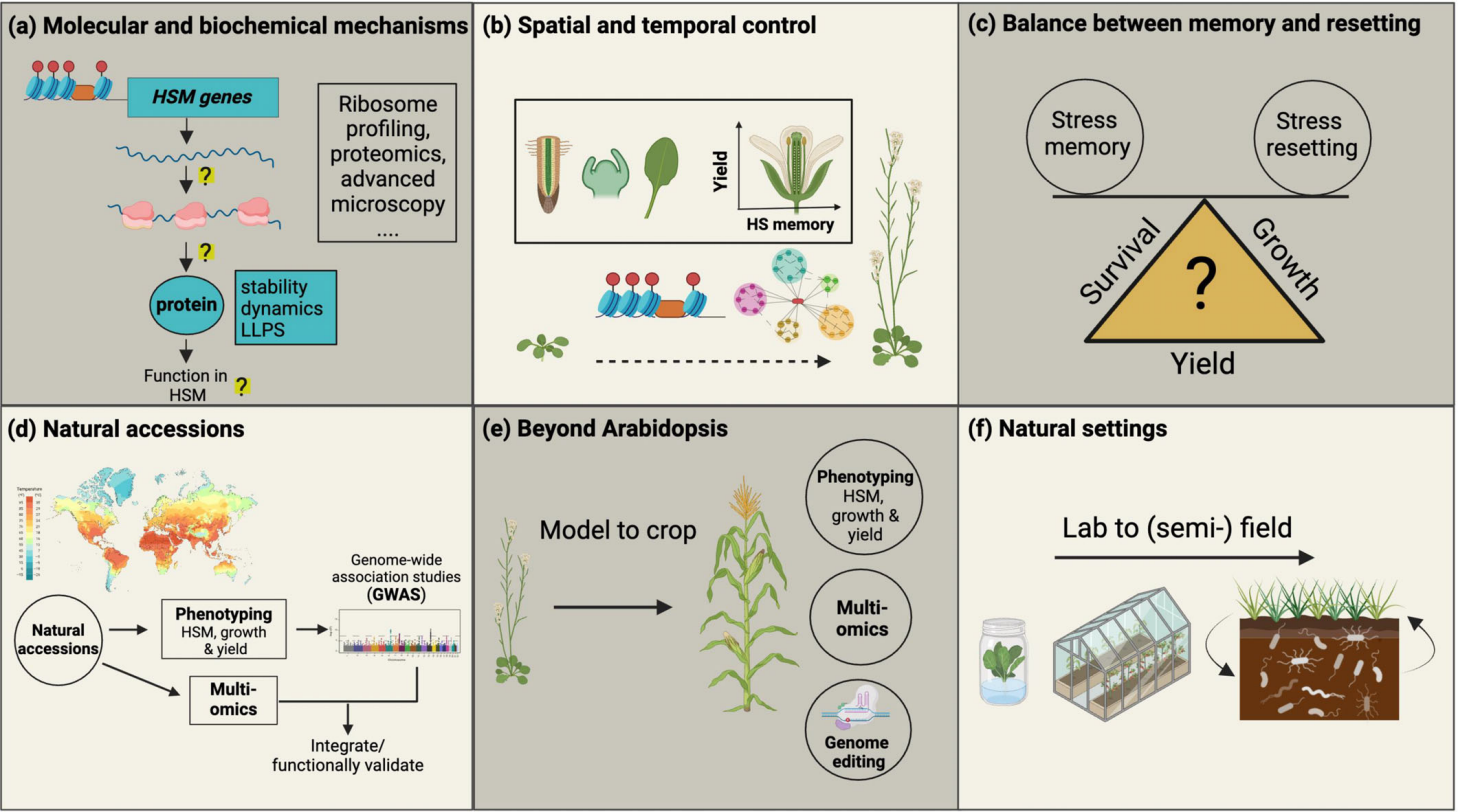
Future research directions for studying heat stress memory (HSM) and resetting in plants. (a) Molecular and biochemical mechanisms to investigate the role of proteins and gene expression in HSM, using advanced techniques like ribosome profiling, proteomics and microscopy. (b) Spatial and temporal control: studying the spatial and temporal dynamics of HSM and its impact on plant productivity and yield. (c) Balance between memory and resetting: understanding the trade‐offs between stress memory and stress resetting in relation to plant survival, growth and yield. (d) Natural accessions: leveraging natural plant accessions and genome‐wide association studies (GWAS) to explore genetic diversity, phenotyping for HSM and integrating multi‐omics data for functional validation. (e) Beyond Arabidopsis: translating findings from model plants like Arabidopsis to crops, with a focus on phenotyping, multi‐omics and genome editing. (f) Natural settings: Extending research from controlled laboratory environments to (semi)‐field and natural conditions to better understand HSM in realistic agricultural settings. HS, heat stress; LLPS, liquid‐liquid phase separation. Question marks indicate processes that need to be studied further in future research. The dotted line in panel (b) indicates the progression through different stages in plant development. Created in BioRender. Bz, S. (2024) https://BioRender.com/r76n900.

The field of HSM has grown substantially in recent years. It is increasingly clear that HSM is controlled at multiple levels, including epigenetic, transcriptional, protein and metabolic mechanisms. Despite progress in the field, several areas require further research to achieve a holistic understanding of HSM and translate these insights into effective crop improvement strategies (Fig. 6).

### 1. Molecular and biochemical mechanisms

Extensive research has significantly advanced our understanding of the transcriptional and epigenetic regulation of HSM in plants, providing valuable insights into how genes with type I or type II transcriptional memory patterns are regulated. Although many genes with transcriptional memory have been identified, knowledge about their roles in memory formation remains fragmentary. Intriguingly, some of these genes, for example *FTSH6*, may not enhance HSM but instead impede it by facilitating resetting mechanisms (Sedaghatmehr *et al*., 2016). Moreover, little is known about which memory‐associated transcripts are translated into functional proteins, highlighting the need for broader investigative approaches, including ribosome profiling and proteomics. Recent findings have highlighted the critical role of protein stabilization under stress and regulated protein degradation during HSM and resetting (Wu *et al*., 2013; Sedaghatmehr *et al*., 2019, 2021; Thirumalaikumar *et al*., 2021), necessitating greater focus in this area and development of improved methods to capture full proteomes at the tissue or single‐cell level. Additionally, there is increasing recognition that LLPS participates in regulating cellular responses, driven by the unique physicochemical and structural properties of proteins. LLPS has emerged as a critical mechanism for sensing elevated temperatures (Jung *et al*., 2020; Bohn *et al*., 2024). Exploring how LLPS affects HSM could reveal new insights into how plants adapt to environmental challenges.

### 2. Spatial and temporal control of stress memory and resetting

Most studies have focused on Arabidopsis seedlings, but it is crucial to extend the research to individual organs and diverse developmental stages. It is already clear that tissues employ different responses to (heat) stress, indicating that the regulatory mechanisms of thermotolerance in vegetative tissues are not identical to those acting in other tissues (Ahsan *et al*., 2010; Fragkostefanakis *et al*., 2016; Olas *et al*., 2021; He *et al*., 2022). Given the high sensitivity of developing reproductive organs to heat and its impact on agricultural yields, it is crucial to understand how HSM is established and regulated in reproductive organs (Agho *et al*., 2025). Identifying alleles that enhance HSM during flowering, pollination, and seed and fruit formation could significantly improve stress resilience and crop productivity. However, studying HSM in reproductive organs, such as flowers and seeds, poses significant technical challenges. These include limitations in accessing specific tissues, varying sensitivities to HS at different developmental stages, and the lack of high‐resolution tools for in‐depth analysis. Addressing these challenges will require advancements in noninvasive imaging, high‐throughput phenotyping, single‐cell‐ or tissue‐specific omics approaches, and the identification and involvement of more suitable model systems.

### 3. Balance between memory and resetting

This is a crucial aspect of plant biology. The benefits of memory must be weighed against costs, such as those required for plant growth or producing seeds and fruits. While several regulators of HSM have been identified, their impact on plant growth and productivity often remains unanalyzed. Identification of the relationship between different stress memory and resetting regulatory modules and their impact on overall plant fitness is essential. A notable example of this complexity is the HSFA2‐FtsH6‐HSP21 module and its connection to autophagy (Fig. 4). Future work should examine the dynamics of these interactions, particularly the optimal length and strength of stress memory, to enhance resilience without compromising overall plant fitness.

### 4. Importance of investigating natural accessions

Although the Arabidopsis reference accession Col‐0 is frequently utilized for consistency across studies, exploration beyond this standard has been limited. An example is accession N13, which exhibits superior HSM compared with Col‐0 due to a knockout mutation in the *FTSH6* gene (Sedaghatmehr *et al*., 2016). Accessions from regions with extreme and variable climates represent promising resources for stress memory research, which could provide insights into its link to growth and generative productivity.

### 5. Beyond Arabidopsis

To advance our understanding of HSM beyond Arabidopsis, it is essential to analyze HS priming and memory mechanisms across a broader range of agriculturally significant species. Currently, physiological and molecular studies on HSM in crop plants remain limited, and research into the conservation or diversification of HSM across species remains scarce. However, studies in rice have revealed conserved roles for the HSP101‐HSA32 module in regulating HSM (Lin *et al*., 2014). The involvement of HSFA2 in thermopriming and regulation of HSM‐related genes has also been observed in tomato and wheat (Fragkostefanakis *et al*., 2016; Guo *et al*., 2020). A recent proteomics study in tall fescue identified hundreds of HSM‐related proteins, some of which are linked to enhanced photosystem II function during repeated HS (Wang *et al*., 2024). With advances in DNA sequencing, cutting‐edge omics technologies and genome editing, crop‐oriented research aiming to enhance stress tolerance through optimized thermopriming and HSM is becoming increasingly feasible and timely.

### 6. Natural settings

Plants in nature do not exist in isolation but rather interact with other plants and numerous bacterial and fungal microorganisms, affecting their responses to environmental stresses (Porter *et al*., 2020). Studies under (near‐) natural environments are crucial for capturing these complex conditions. Evidence shows that root‐colonizing microorganisms significantly affect plant growth and stress survival. Microbes may affect HSM by activating known stress memory regulators or revealing new mechanisms (de Zélicourt *et al*., 2018). Exploring these interactions could uncover novel pathways for maintaining thermotolerance, offering valuable insights for enhancing plant resilience amid climate change.

## Competing interests

None declared.

## Disclaimer

The New Phytologist Foundation remains neutral with regard to jurisdictional claims in maps and in any institutional affiliations.
